# Mortalidade de mulheres com notificação de violência durante a gravidez no Brasil: um estudo caso-controle

**DOI:** 10.1590/0102-311XPT012823

**Published:** 2023-11-10

**Authors:** Marcela Quaresma Soares, Cristiane Magalhães de Melo, Isabella Vitral Pinto, Paula Dias Bevilacqua

**Affiliations:** 1 Instituto René Rachou, Fundação Oswaldo Cruz, Belo Horizonte, Brasil.; 2 Secretaria Municipal de Saúde de Viçosa, Viçosa, Brasil.; 3 Universidade Federal de Viçosa, Viçosa, Brasil.

**Keywords:** Violência de Gênero, Gravidez, Causas de Morte, Homicídio, Gender-Based Violence, Pregnancy, Cause of Death, Homicide, Violencia de Género, Embarazo, Causas de Muerte, Homicidio

## Abstract

Objetivou-se caracterizar as principais causas de óbito de mulheres com notificação de violência interpessoal durante a gravidez e identificar os fatores associados a essas mortes. Trata-se de um estudo caso-controle realizado a partir da análise de dados sobre violência e óbitos ocorridos no Brasil entre 2011 e 2017. Os dados provenientes do Sistema de Informação de Agravos de Notificação e do Sistema de Informação sobre Mortalidade foram analisados por meio da regressão logística múltipla. Os resultados mostraram que 56,4% dos óbitos foram em decorrência de causas externas, sendo 80,1% desses devido ao feminicídio. Identificou-se como fatores de risco associados ao óbito: faixa etária de 30 a 39 anos (OR = 2,53; IC95%: 1,01-6,59); agressão por arma de fogo (OR = 14,21; IC95%: 4,58-31,86) e por objeto perfurocortante (OR = 4,45; IC95%: 1,01-22,73). Como fatores de proteção, observou-se: ser casada/união estável (OR = 0,48; IC95%: 0,24-0,93); ter escolaridade acima de quatro anos (OR = 0,21; IC95%: 0,06-0,63) e residir em municípios com população acima de 100 mil habitantes (OR = 0,23; IC95%: 0,10-0,52). Esta pesquisa foi importante para demonstrar a magnitude do feminicídio entre mulheres com notificação de violência durante a gravidez, assim como as fragilidades na produção de informações sobre as causas externas de óbito no período gravídico-puerperal. Além disso, evidenciou-se os motivos que vulnerabilizam as mulheres para o óbito, reforçando a necessidade urgente do rastreamento pelos profissionais de saúde da violência na gestação.

## Abstract (en)

This study aimed to characterize the main causes of death of women with notification of interpersonal violence during pregnancy and to identify the factors associated with these deaths. This is a case-control study conducted based on relating data on violence to deaths that occurred in Brazil from 2011 to 2017. Data from the Braziliam Information System for Notificable Diseases and the Brazilian Mortality Information System were analyzed using multiple logistic regression. The results showed that 56.4% of the deaths were due to external causes, and 80.1% of which were due to femicide. The following risk factors associated with death were identified: age group from 30 to 39 years (OR = 2.53; 95%CI: 1.01-6.59); firearm assault (OR = 14.21; 95%CI: 4.58-31.86); and by piercing-cutting objects (OR = 4.45; 95%CI: 1.01-22.73). Being married/in a stable union (OR = 0.48; 95%CI: 0.24-0.93); having more than four years of schooling (OR = 0.21; 95%CI: 0.06-0.63); and living in municipalities with a population over 100,000 inhabitants (OR = 0.23; 95%CI: 0.10-0.52) were observed as protective factors. The study was important due to demonstrating the magnitude of femicide among women with notification of violence during pregnancy, as well as the weaknesses in producing information on the external causes of death in the pregnancy-puerperal period. The study also evinced the factors that make women vulnerable to death, reinforcing the urgent need for health professionals to screen for violence during pregnancy.

## Resumen

Se pretende caracterizar las principales causas de muerte de las mujeres por violencia interpersonal durante el embarazo e identificar sus factores asociados. Se trata de un estudio de caso control, realizado a partir de la relación de datos sobre violencia y muertes ocurridas en Brasil entre 2011 y 2017. Los datos del Sistema de Información de Enfermedades de Notificación Obligatoria y del Sistema de Información de Mortalidad se analizaron mediante regresión logística múltiple. Los resultados mostraron que el 56,4% de las muertes se debieron a causas externas, de las cuales el 80,1% fueron feminicidios. Los factores de riesgo asociados con la muerte fueron: grupo de edad de entre 30 y 39 años (OR = 2,53; IC95%: 1,01-6,59), agresión con arma de fuego (OR = 14,21; IC95%: 4,58-31,86) y con objeto perforante (OR = 4,45; IC95%: 1,01-22,73). Respecto a los factores protectores, se observaron los siguientes: estar casada/unión estable (OR = 0,48; IC95%: 0,24-0,93), nivel de estudios superior a cuatro años (OR = 0,21; IC95%: 0,06-0,63) y vivir en municipios con una población superior a 100.000 habitantes (OR = 0,23; IC95%: 0,10-0,52). Este estudio fue clave por mostrar la magnitud del feminicidio entre mujeres con reporte de violencia durante el embarazo, así como las debilidades en la producción de información sobre las causas externas de muerte en el período de embarazo-puerperio. Además, destacó los factores que causan vulnerabilidad a las mujeres para la muerte, lo que refuerza la necesidad de que los profesionales de la salud examinen los casos de violencia durante el embarazo.

## Introdução

A gestação constitui um período da vida que, segundo o imaginário social ocidental, prescrito pela norma patriarcal ^1^ e influenciado pelo cristianismo, sacraliza a maternidade e confere a ela um mito de proteção ^2^^,^^3^. A despeito disso, o período gestacional não isenta as mulheres das violências de gênero as quais são expostas cotidianamente e, por vezes, amplia essa exposição ^4^. Isso porque a gravidez pode aumentar, seja por questões afetivas, seja por questões financeiras, a dependência da mulher, ampliando as chances de sofrerem os mais diversos tipos de violência ^5^^,^^6^.

Embora a violência na gestação seja mais comum do que muitos outros agravos rotineiramente rastreados durante o pré-natal ^4^^,^^7^^,^^8^^,^^9^^,^^10^^,^^11^, esse não é um tema pautado nas consultas que, de modo geral, não abordam o assunto ^12^. No entanto, durante a gravidez, a violência pode trazer consequências significativas para o binômio materno-fetal, como hemorragias, rupturas uterinas, parto prematuro e morte perinatal, além de aumentar o risco de feminicídio e suicídio materno ^6^^,^^7^^,^^8^^,^^9^^,^^13^^,^^14^.

A pesquisa multinacional da Organização Mundial da Saúde (OMS) concluiu que a proporção de violência física na gravidez excedia 5% na grande maioria dos países pesquisados, sendo o menor percentual no Japão (1%) e o maior no Peru (28%) ^15^. Esse achado também foi observado em ampla revisão da literatura acerca do tema, estando presente entre 0,9% e 30% dos casos analisados nos estudos revisados ^9^. Sobre a prevalência da violência por parceiro íntimo na gestação, um estudo conduzido em 19 países apontou uma variação entre 2% na Austrália, Dinamarca, Camboja e Filipinas e 13,5% na Uganda ^10^.

No Brasil, pesquisas evidenciaram prevalências de violências na gravidez (física, psicológica, sexual, entre outras) cometidas por parceiro íntimo de 78,3% no Rio de Janeiro ^16^; 33% em Caxias (Maranhão) ^17^; 31% em Recife (Pernambuco) ^18^; 29,8% em São Luís (Maranhão); 20,1% em Ribeirão Preto (São Paulo) ^19^; 20% em São Paulo ^20^ e 19,1% em Campinas (São Paulo) ^21^. Apesar de altas, essas taxas podem estar subestimadas ^16^^,^^17^^,^^18^^,^^19^^,^^20^^,^^21^.

As diversas taxas de prevalência encontradas na literatura se relacionam em geral às formas ou tipos de violência investigadas, definições empregadas, fontes de dados e métodos de investigação utilizados. É importante salientar que a maioria dos estudos pesquisou a violência por parceiro íntimo, visto que esse é reportado como o principal agressor ^4^^,^^7^^,^^22^. Entretanto, analisar outros vínculos pode ser importante, uma vez que a literatura indica que gestantes adolescentes frequentemente sofrem violência por múltiplos perpetradores ^9^.

Em relação ao feminicídio, um importante estudo caso-controle realizado nos Estados Unidos ^11^ mostrou que mulheres que sofreram violência na gravidez tiveram três vezes a chance de óbito quando comparadas àquelas que não sofreram violência nesse período. Destaca-se que, dentre essas mortes, somente 4,9% ocorreram durante a gestação, sinalizando que a violência na gravidez pode ser um evento sentinela do feminicídio em outros ciclos de vida.

Ainda, estudos têm apontado o homicídio como a principal causa de óbito materno no período gravídico-puerperal nos Estados Unidos ^7^^,^^23^^,^^24^. Estimativas recentes da taxa de mortalidade por homicídio associado à gravidez evidenciaram que o homicídio excedeu todas as principais causas de mortalidade materna em mais de duas vezes no citado país, concluindo que a gravidez e o período pós-parto são momentos de elevado risco de homicídio para mulheres com idade entre 10 e 44 anos ^23^. Embora as taxas de homicídio materno sejam altas nos Estados Unidos, números preocupantes foram encontrados em revisão da literatura global sobre o tema ^14^.

É importante pontuar, entretanto, que os efeitos da violência na saúde materno-fetal podem ser evitáveis. A gestação oferece muitas oportunidades de rastreamento e intervenção precoce no sistema de saúde ^6^^,^^8^, por exemplo, durante o atendimento pré-natal de rotina ou no atendimento episódico no ambiente hospitalar.

Considerando a prevalência da violência na gravidez no Brasil, sua potencialidade para causar mortes e a evitabilidade destas por meio de intervenções do sistema de saúde, o objetivo deste texto foi caracterizar as principais causas de óbito de mulheres com notificação de violência interpessoal durante a gravidez e identificar os fatores associados a essas mortes. Trata-se do primeiro estudo brasileiro a investigar tal associação, contribuindo para melhor compreensão sobre os fenômenos violência e óbito entre mulheres.

Para fins desta pesquisa, considerou-se homicídio como sinônimo de feminicídio, dado o alto índice de violência fatal por questões de gênero descritas na literatura nacional e internacional ^6^^,^^9^^,^^25^^,^^26^. Trata-se de uma postura política que visa contribuir para visibilidade do fenômeno mais extremo da violência de gênero, que é o assassinato associado às relações hierárquicas de poder e discriminação que oprimem e subjugam as mulheres.

## Materiais e métodos

### Delineamento e população do estudo

Trata-se de um estudo observacional analítico, do tipo caso-controle, realizado a partir dos registros de violência interpessoal durante a gravidez e dos registros de óbito dessas mulheres. Foram utilizados dados secundários nacionais, não nominais, provenientes do relacionamento de bancos de dados (*linkage*) realizado pelo Ministério da Saúde. O *linkage* considerou dados do Sistema de Informação de Agravos de Notificação (SINAN), módulo “violências interpessoais e autoprovocadas” referentes ao período de 2011 a 2016 e dados de óbito do Sistema de Informação sobre Mortalidade (SIM), ocorridos entre janeiro de 2011 e setembro de 2017.

O Ministério da Saúde identificou, no período, 812.157 notificações de violências (todos os tipos) contra mulheres de todas as idades no SINAN, enquanto no SIM foram identificados 3.196.446 óbitos de mulheres por todas as causas e idades. Após o *linkage*, foram identificadas, dentre as mulheres com notificação de violência, 17.566 registros de óbito. O detalhamento dos procedimentos realizados no *linkage* foi descrito no livro *Saúde Brasil 2018: Uma Análise de Situação de Saúde e das Doenças e Agravos Crônicos: Desafios e Perspectivas*^27^ e no estudo conduzido por Pinto et al. ^28^.

### Plano amostral

**Figura 1:**
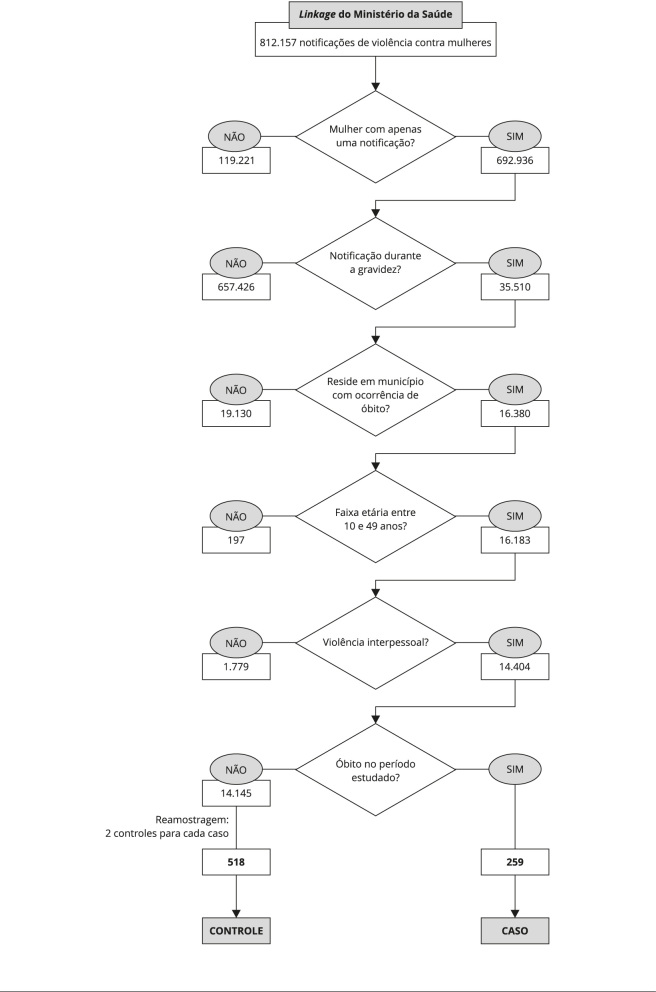
Fluxograma da organização e sistematização dos dados da pesquisa a partir do *linkage* realizado pelo Ministério da Saúde ^27^.

Partindo do banco de dados disponibilizado pelo Ministério da Saúde, como critério de inclusão no estudo considerou-se: (i) mulheres com registro de violência interpessoal; (ii) idade entre 10 e 49 anos; (iii) estar grávida no momento da notificação da violência (Figura 1).

Foram critérios de exclusão no estudo: (i) mulheres com mais de uma notificação de violência; (ii) registro de violência autoprovocada; (iii) não residir em municípios em que ocorreram óbitos (Figura 1).

A exclusão de mulheres que tinham mais de uma notificação no SINAN foi necessária porque as informações para os anos 2015 e 2016 eram preliminares, ou seja, não haviam sido submetidas à rotina de limpeza pelo Ministério da Saúde. Portanto, as duplicidades poderiam superestimar as violências, já que a notificação duplicada seria considerada como um novo episódio.

Como não houve acesso aos dados nominais, para proceder a exclusão acima referida, considerou-se um identificador existente no banco de dados que informava o número de notificações referentes a cada mulher. Ao final desse processo, optou-se por considerar as mulheres com apenas uma notificação de violência no período completo (2011-2016), portanto, todas as mulheres identificadas com mais de uma notificação foram excluídas. Ainda assim, foi possível analisar a violência de repetição a partir dos registros no campo 53 (“ocorreu outras vezes?”), que está presente na ficha de notificação. Tal informação foi utilizada como uma variável explicativa do estudo.

Também foram excluídos os registros de violência autoprovocada, considerando que o objetivo deste estudo foi analisar a violência interpessoal. Para a identificação e exclusão desses registros, realizou-se a análise dos dados relativos ao campo 54 da ficha de notificação do SINAN (“a lesão foi autoprovocada?”), além da categoria aberta “outros” do campo 56 (“tipo de violência”). Nesse último campo, buscou-se registros referentes a agressões autoinfligidas, tentativa de suicídio, autoextermínio ou situações correlatas que não haviam sido assinaladas no campo 54.

Além disso, com o intuito de tornar a amostra mais homogênea, foram excluídas mulheres que não residiam nos municípios em que ocorreram óbitos. O município de residência das mulheres foi identificado através da variável “código do município” na notificação do SINAN.

Após a aplicação dos critérios de inclusão e exclusão referidos, foram definidos os grupos caso e controle. Para o grupo caso foram selecionadas todas as mulheres com notificação de violência interpessoal no SINAN no período de 2011-2016 que tiveram registro de óbito no SIM (Figura 1).

Para o grupo controle, em princípio seriam selecionadas todas as mulheres com notificação de violência interpessoal no SINAN no período de 2011-2016 que não tiveram óbito registrado. Contudo, observou-se um número elevado de mulheres no grupo controle (n = 14.145) em relação ao grupo caso (n = 259) e, uma vez que um número tão elevado não aumentaria o poder estatístico do estudo ^29^, optou-se por uma reamostragem, considerando a proporção de dois controles (n = 518) para cada caso (n = 259) (Figura 1). Para tanto, utilizou-se os procedimentos descritos a seguir.

### Reamostragem

Com o intuito de selecionar um subconjunto de dados com características semelhantes à amostra inicial, ou seja, aquela que melhor representasse a população do estudo, foi empregada a validação cruzada. Tal método estatístico é utilizado para avaliar a variabilidade de um conjunto de dados e a confiabilidade dos modelos treinados, dividindo os dados em dois segmentos: um usado para treinar um modelo e o outro usado para validar e estimar o desempenho desse modelo ^30^^,^^31^.

Na aplicação da validação cruzada, foram geradas aleatoriamente dez amostras de tamanhos iguais baseadas em diferentes seleções dos dados iniciais. Para cada uma dessas amostras geradas foram aplicados os procedimentos de modelagem descritos na análise dos dados.

Para validação e comparação do desempenho dos modelos das amostras obtidas por meio da reamostragem, foi utilizada a métrica AUC (*area under the curve*) ROC (*receiver operating characteristic*). A comparação possibilitou a interpretação da qualidade dos dados, demonstrando a representatividade da amostra e a sensibilidade do modelo à variações ^30^, permitindo a escolha da amostra com melhor desempenho. Assim, foi selecionada a amostra que apresentou a AUC ROC = 0,85, considerada uma discriminação excelente para a métrica ^32^.

### Desfecho de interesse

A variável dicotômica “óbito” (sim, não), sendo esse considerado independentemente do tipo de causa básica. Essa variável teve como fonte de informação o SIM, sendo que as causas básicas foram organizadas em capítulos e agrupamentos, de acordo com a estrutura e princípios da Classificação Internacional de Doenças, 10ª revisão (CID-10) ^33^.

### Variáveis explicativas

Faixa etária em anos (10-19, 20-29, 30-39, 40-49); trimestre gestacional (1º, 2º, 3º); raça/cor (branca, preta/parda/amarela/indígena); escolaridade em anos de estudo (0-4, 5-9, 10-12, > 12); situação conjugal/estado civil (solteira/viúva, casada/união consensual, separada); deficiência/transtorno (não, sim); porte do município de residência em número de habitantes (≤ 100 mil, 101 mil-500 mil, > 500 mil); zona de residência (urbana, rural/periurbana); local de ocorrência (via pública, bar e comércio/serviços, residência, outros); violência de repetição (não, sim); vínculo com o(a) agressor(a) (desconhecido(a), parceiro íntimo, familiar, conhecido(a), outros); tipo de violência (psicológica/moral, física, sexual, outras); meio de agressão (ameaça, força corporal/espancamento, arma de fogo, objeto perfurocortante, outras violências).

Todas as variáveis explicativas foram extraídas do SINAN e categorizadas para permitir melhor visualização dos resultados. As variáveis com campos sem preenchimento (*missing data*) foram agrupadas com os registros informados como “ignorado”.

Para a variável “estado civil”, considerando o baixo número de mulheres viúvas notificadas na amostra (0,3%), optou-se por agregá-las à categoria solteira. Para a variável “raça/cor”, também em razão do baixo número de mulheres declaradas como indígenas e amarelas, para fins de análise estatística, essas foram agrupadas juntamente às pretas e pardas, compondo a categoria “preta/parda/indígena/amarela”. Na análise descritiva dos dados cada raça/cor foi apresentada separadamente.

O “porte do município de residência” foi estabelecido por meio de estimativa da população residente no meio do período, disponível no Departamento de Informática do SUS (DATASUS) ^34^. Tal informação foi combinada a partir do código do município ao banco de dados e, posteriormente, categorizada, considerando o número de habitantes. Na variável “local de ocorrência”, as categorias “residência e habitação coletiva” foram agrupadas, assim como “via pública, bar e comércio/serviços” e na categoria “outros” foram incluídos os registros “escola, local de prática esportiva, indústria e construção”.

Em relação à variável “vínculo com o(a) agressor(a)”, os campos assinalados como “cônjuge”, “ex-cônjuge”, “namorado(a)” e “ex-namorado(a)”, além de vínculos correlatos descritos no campo aberto “outros”, foram considerados como “parceiro íntimo”. As categorias “pai”, “mãe”, “padrasto”, “madrasta”, “filho(a)”, irmão(a)” foram agrupados como familiares. À categoria conhecido(a) foram incluídas: “cuidador(a)”, “patrão/chefe” e “pessoa com relação institucional”. Já à categoria “outros” foi acrescentada “policial/agente da lei”.

As respostas às variáveis “tipo de violência” e “meio de agressão” foram reorganizadas, visto que, no preenchimento dos campos na ficha do SINAN, é possível assinalar mais de uma opção. Para essa reorganização considerou-se como referência o manual *Viva: Instrutivo Notificação de Violência Interpessoal e Autoprovocada*^35^, que orienta que seja assinalado somente o principal tipo de violência perpetrado e meio de agressão empregado, além da proposta de Pinto et al. ^28^ de categorização dessas variáveis a partir de uma hierarquia de importância baseada nas repercussões à saúde.

### Análise dos dados

Inicialmente, foi realizada a descrição das causas de morte e das características gerais dos grupos caso e controle, segundo as variáveis sociodemográficas das mulheres e características da violência.

Após isso, utilizou-se a regressão logística para analisar cada uma das variáveis explicativas em relação ao desfecho de interesse (análise univariada). Em seguida, para a análise múltipla, foram consideradas aquelas variáveis que apresentaram o valor de p < 0,25 na análise anterior. Para a construção do modelo de regressão logística múltipla foi utilizado procedimento de seleção *forward*, permanecendo no modelo variáveis que apresentaram valor de p < 0,05.

Para verificar a intensidade da associação entre as variáveis explicativas e o desfecho, foi estimada a *odds ratio* (OR) com intervalo de 95% de confiança (IC95%). Para avaliação da multicolinearidade das variáveis no modelo ajustado, foi utilizado o teste de fator de inflação da variância (VIF). Por fim, o teste de Hosmer e Lemeshow foi utilizado para verificação da adequação de ajuste do modelo ^32^.

Todas as análises foram realizadas no software R, versão 4.0.3 (http://www.r-project.org), e as interpretações foram feitas considerando o nível de significância de α = 0,05.

### Aspectos éticos

Este estudo integra dois projetos de pesquisa: *Mortalidade de Mulheres Grávidas com Notificação de Violência no Brasil: Avaliação do Risco e Possibilidades de Vigilância*, vinculado ao Instituto René Rachou, Fundação Oswaldo Cruz, e *Como Morrem as Mulheres com Notificação de Violência por Parceiro Íntimo no Brasil?*, vinculado à Universidade Federal de Minas Gerais, ambos aprovados pelos comitês de ética em pesquisa das referidas instituições (CAAE: 37197320.7.0000.5091 e 31334820.5.0000.5149, respectivamente).

## Resultados

Considerando a amostra inicial, das 14.404 mulheres com registro de violência interpessoal na gravidez, 259 (1,8%) morreram no período estudado.

Em relação às causa básicas de óbito, segundo o capítulo da CID-10, observou-se que 56,4% das causas foram classificados no capítulo XX - Causas externas, 10,8% foram classificadas no capítulo XV - Gravidez, parto e puerpério e 6,9% no capítulo I - Algumas doenças infecciosas e parasitárias (Tabela 1).

**Tabela 1:** Causas básicas dos óbitos de mulheres com notificação de violência interpessoal na gravidez, descritas por capítulos e agrupamentos das categorias mais frequentes, de acordo com a estrutura e princípios da Classificação Internacional de Doenças - 10ª revisão (CID-10). Brasil, 2011-2017.

Capítulo da CID-10	n	%
I - Algumas doenças infecciosas e parasitárias *		
Doenças infecciosas intestinais	1	0,4
Tuberculose	1	0,4
Outras doenças bacterianas	1	0,4
Doença pelo vírus da imunodeficiência humana (HIV)	15	5,8
Subtotal	18	6,9
II - Neoplasmas (tumores)	7	2,7
IV - Doenças endócrinas, nutricionais e metabólicas	3	1,2
V - Transtornos mentais e comportamentais	3	1,2
IX - Doenças do aparelho circulatório	16	6,2
X - Doenças do aparelho respiratório	8	3,1
XI - Doenças do aparelho digestivo	16	6,2
XIII - Doenças do sistema osteomuscular e do tecido conjuntivo	1	0,4
XIV - Doenças do aparelho geniturinário	1	0,4
XV - Gravidez, parto e puerpério *		
Gravidez que termina em aborto	5	1,9
Edema, proteinúria e transtornos hipertensivos na gravidez, no parto e no puerpério	5	1,9
Outras afecções obstétricas NCOP	8	3,1
Outras categorias no capítulo XV	10	3,9
Subtotal	28	10,8
XVII - Malformações congênitas, deformidades e anomalias cromossômicas	1	0,4
XVIII - Sintomas, sinais e achados anormais de exames clínicos e de laboratório, NCOP	11	4,2
XX - Causas externas de morbidade e de mortalidade *		
Acidentes	16	6,2
Lesões autoprovocadas intencionalmente	7	2,7
Agressões (feminicídios)	117	45,2
Outras categorias no capítulo XX	6	2,3
Subtotal	146	56,4
Total	259	100,0

NCOP: não classificadas/os em outra parte.

Fonte: Sistema de Informação sobre Mortalidade.

* Capítulos com maior frequência de óbitos e respectivos agrupamentos.

Analisando as causas de acordo com o agrupamento de categorias da CID-10 para cada um dos capítulos descritos anteriormente, observou-se que no capítulo I, o HIV foi responsável pela maior parte dos óbitos (15/18; 83,3%). No capítulo XV, “outras afecções obstétricas” representaram 28,6% (8/28) dos casos e os agrupamentos “gravidez que termina em aborto” e “edema, proteinúria e transtornos hipertensivos” representaram 17,9% (5/28) dos casos, cada (Tabela 1).

No capítulo XX, os feminicídios (nomeados na CID-10 como “agressões”) representaram 80,1% (117/146) dos casos, seguidos dos “acidentes” (16/146; 11%) e “lesões autoprovocadas” (7/146; 4,8%). Destaca-se que do total de óbitos (n = 259), os feminicídios representaram 45,2% (Tabela 1).

Sobre a raça/cor, o agrupamento das categorias preta/parda/amarela/indígena teve um percentual semelhante para os dois grupos, representando 54% no grupo caso e 52% no controle. Como características particulares dos grupos caso e controle, observou-se que, dentre as mulheres com registro de óbito (caso), um alto percentual tinha idade entre 20-29 anos (37,1%) e residiam em municípios com população ≤ 100 mil habitantes (36,8%). Por outro lado, as mulheres sem registro de óbito (controle) tinham, em sua maioria, 10-19 anos (45,5%) e residiam em municípios com mais de 500 mil habitantes (58,6%). Destaca-se que no grupo caso, 12,4% das mulheres tinham menos de quatro anos de estudo, enquanto no grupo controle essa proporção foi de 6%. Além disso, diferentemente do esperado, enquanto no grupo caso 30,1% das mulheres tinham registro de violência de repetição, no grupo controle esse percentual foi de 40,4% (Tabela 2).

**Tabela 2:** Distribuição dos grupos caso e controle, segundo as variáveis sociodemográficas das mulheres e características da violência. Brasil, 2011-2017.

Variáveis	Caso (n = 259)	Controle (n = 14.145)
%	%
Faixa etária (anos)		
10-19	27,3	45,5
20-29	37,1	35,2
30-39	29,0	16,1
40-49	6,6	3,2
Trimestre gestacional		
Primeiro	37,8	34,8
Segundo	32,4	32,3
Terceiro	19,3	25,4
Ignorado	10,5	7,5
Raça/Cor		
Branca	34,4	37,0
Preta	13,5	10,6
Parda	38,2	39,3
Amarela	1,9	1,3
Indígena	0,4	0,8
Ignorado	11,6	11,0
Escolaridade (anos de estudo)		
0-4	12,4	6,0
5-9	30,5	35,2
10-12	18,0	24,4
> 12	4,1	5,3
Ignorado	35,0	29,1
Situação conjugal/Estado civil		
Solteira/Viúva	45,9	48,7
Casada/União consensual	35,7	34,6
Separada	3,4	3,4
Ignorado	15,0	13,3
Deficiência/Transtorno		
Não	72,6	80,1
Sim	7,5	3,7
Ignorado	19,9	16,2
Zona de residência		
Urbana	85,7	92,5
Periurbana/Rural	10,2	4,4
Ignorado	4,1	3,1
Porte do município (habitantes)		
≤ 100 mil	36,8	6,7
101 mil-500 mil	35,7	34,7
> 500 mil	27,5	58,6
Local de ocorrência		
Via pública, bares ou comércio/serviços	24,3	16,6
Residência/Habitação coletiva	52,5	61,2
Outros	8,5	7,2
Ignorado	14,7	15,0
Violência de repetição		
Não	39,8	34,6
Sim	30,1	40,4
Ignorado	30,1	25,0
Vínculo com o(a) agressor(a)		
Desconhecido(a)	13,5	10,5
Parceiro íntimo	46,3	50,4
Familiar	9,3	15,2
Conhecido(a)	11,2	9,1
Outros	3,5	5,3
Ignorado	16,2	9,5
Tipo de violência *		
Psicológica/Moral	2,3	4,9
Física	69,1	48,5
Sexual	14,7	28,9
Outras	10,4	15,1
Ignorado	3,5	2,6
Meio de agressão **		
Ameaça	2,7	5,0
Força corporal/Espancamento	52,1	52,5
Arma de fogo	16,6	3,4
Objeto perfurocortante	8,5	2,2
Outras violências	7,7	8,5
Ignorado	12,4	28,4

Fonte: Sistema de Informação de Agravos de Notificação.

* Principal tipo de violência;

** Principal meio de agressão.

Houve um importante percentual de respostas assinaladas como “ignorado”, evidenciando a extensão da incompletude (percentual de registros ou campos não preenchidos) dos dados do SINAN. Em relação ao preenchimento das características sociodemográficas das mulheres, as variáveis raça/cor, situação conjugal/estado civil, deficiência/transtorno tiveram um grau de incompletude regular (10% a 20%), enquanto escolaridade apresentou um grau de incompletude ruim (20% a 50%), conforme o escore proposto por Romero & Cunha ^36^. Sobre as características das violências notificadas, com exceção de “tipo de violência”, todas as variáveis apresentaram percentual expressivo na categoria “ignorado”, com incompletude variando entre regular e ruim ^36^, com destaque para a variável “violência de repetição” (Tabela 2).

Com ajuda da análise múltipla, as seguintes variáveis foram associadas ao óbito: “faixa etária”, “escolaridade”, “situação conjugal/estado civil”, “porte do município de residência” e “meio de agressão” (Tabela 3).

**Tabela 3:** Fatores de risco para óbitos de mulheres com notificação de violência interpessoal durante a gravidez. Brasil, 2011 a 2017.

Variáveis	Análises univariadas		Análise múltipla	
	OR (IC95%)	Valor de p	OR (IC95%)	Valor de p
Faixa etária (anos)				
10-19	Referência		Referência	
20-29	1,51 (0,98-2,32)	0,061	1,39 (0,64-3,11)	0,360
30-39	2,88 (1,75-4,76)	< 0,001	2,53 (1,01-6,59)	0,031
40-49	2,84 (1,24-6,45)	0,012	1,98 (0,23-11,25)	0,180
Trimestre gestacional				
Primeiro	Referência		-	-
Segundo	0,77 (0,50-1,20)	0,248	-	-
Terceiro	0,57 (0,35-0,94)	0,028	-	-
Raça/Cor			-	-
Branca	Referência			
Preta/Parda/Amarela/Indígena	1,18 (0,80-1,74)	0,412	-	-
Escolaridade (anos de estudo)				
0-4	Referência		Referência	
5-9	0,23 (0,10-0,51)	< 0,001	0,21 (0,06-0,63)	0,009
10-12	0,27 (0,12-0,61)	< 0,001	0,24 (0,07-0,73)	0,001
> 12	0,25 (0,08-0,74)	0,016	0,22 (0,04-0,94)	0,048
Situação conjugal/Estado civil				
Solteira/Viúva	Referência		Referência	
Casada/União consensual	0,68 (0,44-1,02)	0,067	0,48 (0,24-0,93)	0,040
Separada	0,67 (0,21-1,84)	0,459	0,16 (0,01-1,06)	0,153
Deficiência/Transtorno				
Não	Referência			
Sim	2,11 (0,93-4,78)	0,711	-	-
Porte do município (habitantes)				
≤ 100 mil	Referência		Referência	
101 mil-500 mil	0,21 (0,13-0,56)	< 0,001	0,23 (0,10-0,52)	< 0,0001
> 500 mil	0,08 (0,05-0,01)	< 0,001	0,06 (0,03-0,15)	< 0,0001
Zona de residência				
Urbana	Referência		-	-
Periurbana/Rural	2,58 (1,31-5,11)	0,006	-	-
Local de ocorrência				
Via pública, bares ou comércio/serviços	Referência		-	-
Residência/Habitação coletiva	0,41 (0,25-0,66)	< 0,001	-	-
Outros	0,62 (0,28-1,34)	0,233	-	-
Violência de repetição				
Não	Referência		-	-
Sim	0,65 (0,42-1,01)	0,055	-	-
Vínculo com o (a) agressor(a)				
Desconhecido(a)	Referência			
Parceiro íntimo	0,66 (0,37-1,20)	0,163	-	-
Familiar	0,53 (0,25-1,12)	0,098	-	-
Conhecido(a)	1,19 (0,55-2,55)	0,659	-	-
Outros	0,66 (0,24-1,71)	0,403	-	-
Tipo de violência *				
Psicológica/Moral	Referência			
Física	1,91 (0,74-5,89)	0,021	-	-
Sexual	0,95 (0,33-3,22)	0,938	-	-
Outras	1,98 (0,51-8,18)	0,346	-	-
Meio de agressão **				
Ameaça	Referência		Referência	
Força corporal/Espancamento	1,21 (0,51-3,20)	0,680	1,65 (0,49-6,73)	0,410
Arma de fogo	11,42 (3,65-30,64)	< 0,001	14,21 (4,58-31,86)	< 0,001
Objeto perfurocortante	4,44 (1,62-13,35)	0,005	4,45 (1,01-22,73)	0,003
Outras violências	1,28 (0,49-3,67)	0,624	2,76 (0,71-12,68)	0,177

IC95%: intervalo de 95% de confiança; OR: *odds ratio*.

Fonte: Sistema de Informação de Agravos de Notificação e Sistema de Informação sobre Mortalidade.

* Principal tipo de violência;

** Principal meio de agressão.

Observou-se como fatores de risco: idade entre 30-39 anos, uma vez que mulheres nessa faixa etária tiveram 2,53 (IC95%: 1,01-6,59) vezes a chance de óbito quando comparadas às mulheres na faixa etária entre 10-19 anos; agressão por arma de fogo ou por objetos perfurocortantes, já que as chances de óbito por esses meios foram, respectivamente, 14,21 (IC95%: 4,58-31,86) e 4,45 (IC95%: 1,01-22,73) vezes a chance de óbito em comparação à agressão por ameaça (Tabela 3).

Como fatores de proteção foram identificados: ser casada/união consensual (OR = 0,48; IC95%: 0,24-0,93), quando comparada a categoria solteira/viúva, ter escolaridade superior a quatro anos (OR variando entre 0,21; IC95%: 0,06-0,63 e 0,24; IC95%: 0,07-0,73) e residir em municípios com população superior a 100 mil habitantes (OR = 0,23; IC95%: 0,10-0,52 e OR = 0,06; IC95%: 0,03-0,15) (Tabela 3).

## Discussão

Os resultados deste estudo apontaram para um número expressivo de mortes por causas externas, sobretudo por feminicídios, que representaram 45,2% do total de óbitos. Os fatores de risco para o óbito de mulheres com notificação de violência durante a gravidez foram: idade entre 30-39 anos e/ou ter sofrido agressão por meio do uso de arma de fogo ou por objeto perfurocortante. Ainda, os resultados mostraram que ser solteira/viúva, com baixa escolaridade e/ou residir em municípios até 100 mil habitantes também se constituíram fatores de risco para os óbitos, considerando que ser casada/união estável, com escolaridade acima de quatro anos e/ou residir em municípios com população > 100 mil habitantes apresentaram-se como fatores de proteção.

No Brasil, observou-se uma tendência de crescimento da mortalidade de mulheres em idade fértil por causas externas em todas as regiões ^37^. No período entre 2011 e 2017, as causas externas e feminicídios constituíram, respectivamente, 18,3% e 5,8% dos óbitos de mulheres em idade fértil ^38^. Apesar de impactantes, tais dados são muito inferiores aos apresentados neste estudo, cujo número de mortes por causas externas foi exorbitante, tendo, somente os feminicídios representado quase metade dos óbitos de mulheres que tiveram notificação de violência interpessoal na gravidez.

Dados do Observatório Obstétrico Brasileiro ^39^ evidenciaram a magnitude das causas externas na mortalidade de gestantes e puérperas. Enfatiza-se que tais mortes não são consideradas no cálculo da razão da mortalidade materna ^5^^,^^33^^,^^39^^,^^40^, embora a gestação seja considerada um fator de risco para o óbito ^14^. Considerando, assim, a expressividade do feminicídio entre mulheres que sofreram violência na gravidez e a possibilidade de vincular esses casos a um histórico de violência na vida e na gestação, reitera-se que tais mortes poderiam ser evitadas.

Em relação às causas de óbito classificadas no capítulo XV (Gravidez, parto e puerpério), esta pesquisa mostrou que a maior parte dessas foi relacionada a “outras afecções obstétricas”. Evidencia-se que essas mortes, assim como as demais listadas no mesmo capítulo, podem estar relacionadas às causas violentas, entretanto não foi possível analisá-las. Apesar do Ministério da Saúde ^41^ utilizar, desde 2013, códigos marcadores que associam as causas externas às mortes ocorridas no período gravídico puerperal ^5^^,^^40^, esses não são utilizados como causa básica de óbito, visto que não são adotados pela CID-10 ^33^^,^^42^.

Assim, observamos que a produção de dados fidedignos quanto ao feminicídio no período gravídico-puerperal ainda é um desafio, tal situação contribui para a invisibilidade das violências nos protocolos voltados para a saúde materno-infantil, políticas de enfrentamento às desigualdades de gênero e propostas de vigilância do óbito materno ^5^. No que diz respeito às informações sobre as mortes violentas, é necessário o aprimoramento das ações de vigilância por meio da incorporação de dados que melhor identifiquem os feminicídios, contribuindo para a qualificação das análises e possibilitando a identificação de fatores potencialmente evitáveis ^25^.

Os resultados também mostraram que a “gravidez que termina em aborto” foi a segunda causa de óbito materno entre mulheres que sofreram violência. Considerando que a violência é um fator de risco para o abortamento ^9^^,^^13^ e, que no Brasil a prática é criminalizada na maioria dos casos, a exposição das mulheres a práticas inseguras as sujeitam a situações de risco de morte, seja pela precariedade dos meios utilizados, seja pela demora em buscar atendimento em casos de intercorrências decorrentes do procedimento.

Considerando as doenças infecciosas, o HIV também foi descrito como associado à violência ^43^^,^^44^. O óbito decorrente de tal infecção também pode ser relacionado aos efeitos negativos da violência nos cuidados de saúde que demandam acompanhamento contínuo ^6^^,^^28^.

Sobre a faixa etária, algumas hipóteses poderiam contribuir para explicar o fato de mulheres com idade entre 30-39 anos terem maior chance de óbito. Uma delas é que a mortalidade geral de mulheres em idade fértil é diretamente proporcional ao aumento da idade, embora seja mais frequente entre 40-49 anos ^37^^,^^38^. No mesmo sentido, identifica-se que a idade se constitui como um fator risco para a mortalidade por causas obstétricas ^45^, que neste estudo, apresentaram-se como a segunda causa de morte identificada. Por fim, considerando que a violência de gênero tende a se agravar ao longo do tempo, culminando com casos graves e até o feminicídio, a exposição prolongada e a recorrência das diversas violências podem contribuir para o agravamento do desfecho.

Importante observar, contudo, que nesta pesquisa a violência de repetição não foi estatisticamente associada ao óbito. Porém, essa análise pode ter sido prejudicada em função da decisão metodológica de exclusão das mulheres que tiveram mais de uma notificação de violência na amostra selecionada e pela elevada incompletude das respostas para essa variável.

O estado civil solteira/viúva se apresentou neste estudo como um fator de risco para o óbito, corroborando a literatura que aponta que há uma maior proporção de óbitos entre solteiras, sejam elas mulheres em idade fértil (óbito por todas as causas) ^26^^,^^37^, sejam elas gestantes e puérperas (óbito por agressão) ^24^^,^^40^^,^^46^. O fato de uma mulher ter se separado de um parceiro abusivo também tem sido associado ao feminicídio ^6^^,^^47^. Nesse sentido, é importante refletir que muitas das mulheres declaradas como solteiras podem viver ou terem saído de relacionamentos não oficializados sem efeito civil, o que mostra a fragilidade da avaliação baseada na situação conjugal/estado civil, considerando os arranjos complexos das relações afetivas/sexuais.

A escolaridade inferior a quatro anos foi fator de risco para a ocorrência do óbito, isso ocorre porque mulheres com níveis educacionais mais altos geralmente apresentam maior autoconfiança, habilidades no uso de informações e recursos, rede de apoio e autonomia financeira, conferindo a elas maiores recursos para reconhecer e romper o ciclo de violência em relacionamentos abusivos. Esse resultado reforça a importância da escolarização de meninas e mulheres na prevenção da violência ^28^.

O maior porte do município como um fator de proteção se dá possivelmente devido a maior disponibilidade e organização da rede de serviços relacionados ao enfrentamento da violência contra as mulheres ofertados nessas localidades ^27^^,^^28^. Os serviços especializados para atendimento às situações de violência contra as mulheres abrangem menos de 20% dos municípios brasileiros e concentram-se, sobretudo nas capitais, o que dificulta o acesso das residentes em municípios de menor porte, tornando-as mais vulneráveis à violência e ao feminicídio ^48^.

Além da menor disponibilidade de serviços especializados, é importante refletir sobre como o sistema social baseado na cultura patriarcal se faz presente na vida das mulheres, contribuindo para naturalizar e legitimar as diversas formas de violência. Esse sistema que oprime e controla as mulheres atravessa a vida de todas, porém, para aquelas residentes em municípios de menor porte, a fragilidade das redes de apoio, bem como a hegemonia do discurso patriarcal podem dificultar a resistência e a subversão, mantendo-as em relações abusivas para preservar uma suposta ideia de instituição familiar ^1^^,^^49^^,^^50^.

Sobre os meios de agressão, a arma de fogo e objeto perfurocortante constituíram-se como importantes fatores associados ao óbito, resultado esperado devido à maior letalidade desses meios. Tal achado reforça a necessidade de políticas que visem a redução da violência armada, como leis restritivas de porte ^22^^,^^24^^,^^47^^,^^51^^,^^52^, o que é contrário ao que ocorreu no Brasil, em que a discussão sobre a flexibilização do acesso às armas de fogo ganhou evidência com a ascensão de grupos políticos adeptos da pauta armamentista. Desde 2017, houve um crescimento de 100,6% no total de registro de posses de arma e, desde 2019, já foram editados mais de trinta atos normativos com o objetivo de ampliar o acesso da população às armas e munições. Além disso, houve uma deterioração dos mecanismos de controle de armas ilegais ^51^^,^^52^. Trata-se de um cenário preocupante que pode gerar um número ainda maior de feminicídios e outros crimes violentos.

Este estudo apresentou algumas limitações, entre elas destaca-se aquelas relativas ao uso de dados secundários como: subnotificação de casos (muito frequente na violência interpessoal); falta de padronização na coleta de dados e ausência de informações, resultante do elevado volume de campos com preenchimento ignorado. Os problemas na qualidade dos registros dos dados podem ter enviesados resultados de determinadas categorias de variáveis, podendo ser, inclusive, o motivo da não associação de algumas variáveis que são sabidamente relacionadas à violência, como a raça/cor, a violência por parceiro íntimo e a violência de repetição. Essas limitações indicam a necessidade de sensibilização e capacitação de profissionais sobre a importância da notificação e da qualidade no preenchimento das fichas.

Outra limitação se relaciona ao menor número de ocorrência em algumas categorias das variáveis analisadas, fato inerente ao agravo, o que pode ter contribuído para a maior amplitude de alguns dos intervalos de confiança. Ainda, é importante considerar que, por se tratarem de informações nacionais, há heterogeneidade dos dados, assim, sugerimos a realização de outros estudos cujas análises partam de dados regionais.

Por outro lado, por utilizar dados secundários, algumas limitações comuns ao desenho caso-controle foram minimizadas, configurando-se como forças. Dentre elas, destaca-se o viés da memória, já que os dados sobre a exposição já estavam coletados. Também, no caso do viés de seleção, casos e controles foram provenientes da mesma amostra, ou seja, mulheres na mesma faixa etária, com violência interpessoal notificada na gravidez, que residiam nos mesmos municípios. Além disso, há a possibilidade de assegurar a sequência dos eventos (violência e óbito) e, considerando a baixa incidência do desfecho de interesse, a odds ratio fornece uma boa estimativa do risco relativo.

Por fim, ressalta-se que os resultados apresentados permitem a identificação de fatores que vulnerabilizam as mulheres para o óbito que devem ser considerados na proposição e avaliação das ações de enfrentamento às violências. Indicam também a necessidade de um olhar mais atento aos municípios de menor porte, por intermédio do desenvolvimento de pesquisas que busquem compreender a realidade das violências nessas localidades e, também, da implementação das redes de proteção à mulher.

Esta investigação foi importante, ainda, para evidenciar a magnitude do feminicídio entre mulheres com notificação de violência durante a gestação, assim como as fragilidades na produção da informações sobre as causas externas de óbito no período gravídico-puerperal e, sobretudo, para reforçar a necessidade urgente da inclusão do rastreamento da violência nos protocolos de assistência ao pré-natal, parto e puerpério, a fim de detectar precocemente os casos, com vistas a contribuir para redução dos desfechos letais.

## References

[1] Narvaz MG, Koller SH (2006). Famílias e patriarcado da prescrição normativa à subversão
criativa. Psicol Soc.

[2] Assis ACM, Muneratto BG (2015). Gení apedrejada, Madalena arrependida e Maria santificada
relações entre a misoginia no imaginário cristão e o respaldo ideológico na
perpetuação da violência contra a mulher. Revista Diálogos Acadêmicos.

[3] Gonzaga PRB, Mayorga C (2019). Violências e instituição maternidade uma reflexão feminista
decolonial. Psicol Ciênc Prof.

[4] Ribeiro MRC, Pessoa BPT, Sauaia GA, Schraiber LB, Queiroz RCS, Batista RFL (2020). Violência contra mulheres antes e durante o período gestacional
diferenças em taxas e perpetradores. Rev Bras Saúde Mater Infant.

[5] Alves SV, Antunes MBC (2009). Morte por causas externas durante o período gravídico-puerperal
como classificá-las?. Cad Saúde Colet (Rio J.).

[6] World Health Organization (2011). Intimate partner violence during pregnancy.

[7] Campbell J, Matoff-Stepp S, Velez ML, Cox HH, Laughon K (2021). Pregnancy-associated deaths from homicide, suicide, and drug
overdose review of research and the intersection with intimate partner
violence. J Womens Health (Larchmt).

[8] Alhusen JL, Ray E, Sharps P, Bullock L (2015). Intimate partner violence during pregnancy maternal and neonatal
outcomes. J Womens Health (Larchmt).

[9] Taillieu TL, Brownridge DA (2010). Violence against pregnant women prevalence, patterns, risk
factors, theories, and directions for future research. Aggress Violent Behav.

[10] Devries KM, Kishor S, Johnson H, Stöckl H, Bacchus LJ, Garcia-Moreno C (2010). Intimate partner violence during pregnancy analysis of prevalence
data from 19 countries. Reprod Health Matters.

[11] McFarlane J, Campbell JC, Sharps P, Watson K (2002). Abuse during pregnancy and femicide urgent implications for
women's health. Obstet Gynecol.

[12] Garnweidner-Holme LM, Lukasse M, Solheim M, Henriksen L (2017). Talking about intimate partner violence in multi-cultural
antenatal care a qualitative study of pregnant women's advice for better
communication in South-East Norway. BMC Pregnancy Childbirth.

[13] Stöckl H, Filippi V, Watts C, Mbwambo JKK (2012). Induced abortion, pregnancy loss and intimate partner violence in
Tanzania a population based study. BMC Pregnancy Childbirth.

[14] Cliffe C, Miele M, Reid S (2019). Homicide in pregnant and postpartum women worldwide a review of
the literature. J Public Health Policy.

[15] Garcia-Moreno C, Jansen HAFM, Ellsberg M, Heise L, Watts CH, WHO Multi-country Study on Women's Health and Domestic Violence
against Women Study Team (2006). Prevalence of intimate partner violence findings from the WHO
multi-country study on women's health and domestic violence. Lancet.

[16] Moraes CL, Reichenheim ME (2002). Domestic violence during pregnancy in Rio de Janeiro,
Brazil. Int J Gynaecol Obstet.

[17] Conceição HN, Coelho SF, Madeiro AP (2021). Prevalência e fatores associados à violência por parceiro íntimo
na gestação em Caxias, Maranhão, 2019-2020. Epidemiol Serv Saúde.

[18] Silva EP, Ludermir AB, Araújo TVB, Valongueiro SA (2011). Frequência e padrão da violência por parceiro íntimo antes,
durante e depois da gravidez. Rev Saúde Pública.

[19] Ribeiro MRC, Silva AAM, Schraiber LB, Murray J, Alves MTSSB, Batista RFL (2020). Inversion of traditional gender roles and intimate partner
violence against pregnant women. Cad Saúde Pública.

[20] Durand JG, Schraiber LB (2007). Violência na gestação entre usuárias de serviços públicos de
saúde da Grande São Paulo prevalência e fatores associados. Rev Bras Epidemiol.

[21] Audi CAF, Segall-Corrêa AM, Santiago SM, Andrade MGG, Pèrez-Escamila R (2008). Violence against pregnant women prevalence and associated
factors. Rev Saúde Pública.

[22] Stöckl H, Devries K, Rotstein A, Abrahams N, Campbell J, Watts C (2013). The global prevalence of intimate partner homicide a systematic
review. Lancet.

[23] Wallace M, Gillispie-Bell V, Cruz K, Davis K, Vilda D (2021). Homicide during pregnancy and the postpartum period in the United
States, 2018-2019. Obstet Gynecol.

[24] Cheng D, Horon IL (2010). Intimate-partner homicide among pregnant and postpartum
women. Obstet Gynecol.

[25] World Health Organization Femicide..

[26] Meneghel SN, Hirakata VN (2011). Femicídios homicídios femininos no Brasil. Rev Saúde Pública.

[27] Ministério da Saúde (2019). Saúde Brasil 2018: uma análise de situação de saúde e das doenças e
agravos crônicos: desafios e perspectivas..

[28] Pinto IV, Bernal RTI, Souza MFM, Malta DC (2021). Fatores associados ao óbito de mulheres com notificação de
violência por parceiro íntimo no Brasil. Ciênc Saúde Colet.

[29] Szklo M, Nieto FJ (2018). Epidemiology: beyond the basics..

[30] Refaeilzadeh P, Tang L, Liu H, Liu L, Özsu MT (2009). Encyclopedia of database systems.

[31] Laboratório de Estatística e Geoinformação Métodos de reamostragem..

[32] Hosmer DW, Lemeshow S, Sturdivant RX (2013). Applied logistic regression..

[33] Organização Mundial da Saúde (2008). CID-10 - Classificação Estatística Internacional de Doenças e Problemas
Relacionados a Saúde. 10ª Revisão - manual de instruções..

[34] Departamento de Informática do SUS Projeção da população das Unidades da Federação por sexo e grupos de
idade: 2000-2030..

[35] Ministério da Saúde (2016). Viva: instrutivo notificação de violência interpessoal e
autoprovocada..

[36] Romero DE, Cunha CB (2006). Avaliação da qualidade das variáveis sócio-econômicas e
demográficas dos óbitos de crianças menores de um ano registrados no Sistema
de Informações sobre Mortalidade do Brasil (1996/2001). Cad Saúde Pública.

[37] Souza AMG (2019). Avaliação da mortalidade de mulheres em idade fértil vítimas de
violência.

[38] Departamento de Informática do SUS Mortalidade - Brasil, 2011 a 2017..

[39] Rodrigues AS, Francisco RPV, Herzog RS (2022). Observatório Obstétrico Brasileiro apresenta dados inéditos de
mortalidade materna no Brasil.

[40] Alves MMR, Alves SV, Antunes MBC, Santos DLP (2013). Causas externas e mortalidade materna proposta de
classificação. Rev Saúde Pública.

[41] Ministério da Saúde (2013). Protocolo de codificações especiais em mortalidade.

[42] World Health Organization ICD-11. Reference guide..

[43] Barros C, Schraiber LB, França-Junior I (2011). Associação entre violência por parceiro íntimo contra a mulher e
infecção por HIV. Rev Saúde Pública.

[44] Ceccon RF, Meneghel SN (2015). HIV e violência contra mulheres estudo em município com alta
prevalência de aids no Sul do Brasil. Rev Panam Salud Pública.

[45] Bitencourt S, Dias MAB, Wakimoto MD (2013). Vigilância do óbito materno, infantil e fetal e atuação em comitês de
mortalidade.

[46] Spence S Two pink lines: exploring Florida's pregnancy-associated intimate
partner homicides..

[47] Campbell JC, Webster D, Koziol-McLain J, Block C, Campbell D, Curry MA (2003). Risk factors for femicide in abusive relationships results from a
multisite case control study. Am J Public Health.

[48] Campos CH (2015). Desafios na implementação da Lei Maria da Penha. Revista Direito GV.

[49] Saffioti H (2015). Gênero, patriarcado, violência..

[50] Saffioti HIB (2001). Contribuições feministas para o estudo da violência de
gênero. Cadernos Pagu.

[51] Cerqueira D, Ferreira H, Bueno S, Alves PP, Lima RS, Marques D (2021). Atlas da violência.

[52] Fórum Brasileiro de Segurança Pública (2021). Anuário brasileiro de segurança pública, 2020.

